# How to choose an evidence-based medicine knowledge test for medical students? Comparison of three knowledge measures

**DOI:** 10.1186/s12909-018-1391-z

**Published:** 2018-12-04

**Authors:** Ivan Buljan, Ana Jerončić, Mario Malički, Matko Marušić, Ana Marušić

**Affiliations:** Department of Research in Biomedicine and Health, University of Split School of Medicine, Šoltanska 2, 21000 Split, Croatia

**Keywords:** Evidence-based medicine, ACE test, Berlin test, Fresno test, Knowledge assessment, Medical students

## Abstract

**Background:**

There are a few studies of alignment between different knowledge-indices for evidence-based medicine (EBM). The aim of this study was to investigate whether the type of test used to assess knowledge of EBM affects the estimation of this knowledge in medical students.

**Methods:**

Medical students enrolled in 1-week EBM course were tested with the Fresno, Berlin, and ACE tests at the beginning and the end of the course. We evaluated the ability of these tests to detect a change in the acquired level of EBM knowledge and compared the estimates of change with those of the Control group that was tested with the ACE and Berlin tests before and after an unrelated non-EBM course. The distributions of test scores and average item difficulty indices were compared among the tests and the groups.

**Results:**

Test scores improved on all three tests when compared with their pre-test results and the control. Students had on average a “good” performance on the ACE test, “sufficient” performance on the Berlin test, and “insufficient” performance or have “not passed” on the Fresno test. The post-test improvements in performance on the Fresno test (median 31% increase in percent scores, 95% confidence interval (CI) 25–42%) outperformed those on the ACE (13, 95% CI 13–20%) and Berlin tests (13, 95% CI 7–20%). Post-test score distributions demonstrated that the ACE test had less potential to discriminate between levels of EBM knowledge than other tests.

**Conclusion:**

The use of different EBM tests resulted in different assessment of general EBM knowledge in a sample of graduate medical students, with lowest results on the Fresno and highest on the ACE test. In the light of these findings, EBM knowledge assessment should be based on the course’s content and learning objectives.

**Electronic supplementary material:**

The online version of this article (10.1186/s12909-018-1391-z) contains supplementary material, which is available to authorized users.

## Background

Evidence-based medicine (EBM) is a “conscientious, explicit and judicious use of current best evidence in making decisions about the care of an individual patient” [1]. It is accepted as an empirically grounded approach to health care [2], and as a framework for diagnosis and treatment of most health conditions [3]. The Sicily Statement on evidence-based practice emphasizes that all health-care professionals should understand EBM principles, recognize EBM in action and apply best available evidence in order to more easily provide best practice [4].

EBM teaching is recommended as an essential part of medical/clinical education [5], and the World Federation for Medical Education recommends that scientific method and EBM must be taught in medical school curricula to enable students’ preparation for professional life [6]. However, there is little evidence for success of EBM educational programs and transfer of acquired knowledge to clinical practice [7]. Practitioners often have little time to keep up with new research results and guidelines which could be implemented in practice [8, 9].

There is a large body of research on the effectiveness of different EBM educational interventions, but only a small number of studies had used the same measures to assess EBM knowledge [7]. The two most commonly used tests, Fresno [10] and Berlin [11], were used as an assessment tool of undergraduate EBM competency in fewer than a dozen studies so far [10–19]. In the study by West et al. in 2011, both the Fresno and Berlin tests were applied together, to show that there was generally a significant increase in knowledge on both tests after EBM education [19]. However, more in-depth comparison between the results on these two tests was not reported. The study by Lai et al. showed no significant correlation between the Berlin test and adapted Fresno test [16]. In 2014, the ACE (Assessing Competency in Evidence Based Medicine) Tool was developed, with reported high reliability and validity for measuring EBM knowledge of medical undergraduates [14].

The primary objective of our study was to investigate whether the type of test used to assess students’ EBM knowledge affected the estimation of this knowledge. For this purpose, we used all three tests – Fresno [10], Berlin [11] and ACE [14], and tested them in a sample of third-year medical students. We also investigated whether knowledge indices were sensitive to the change in the acquired level of knowledge by comparing the improvements in knowledge indices of students attending the EBM course to those who did not attend the course. The testing was performed during an EBM course delivered at the third year of graduate medical studies as a part of the vertically integrated undergraduate course on research methodology and EBM [20, 21].

## Methods

### Setting

The research was conducted at the University of Split School of Medicine (USSM) in November 2015 and June–July 2016. We used the quasi-experimental controlled before and after study design.

### Course details

**Fig. 1:**
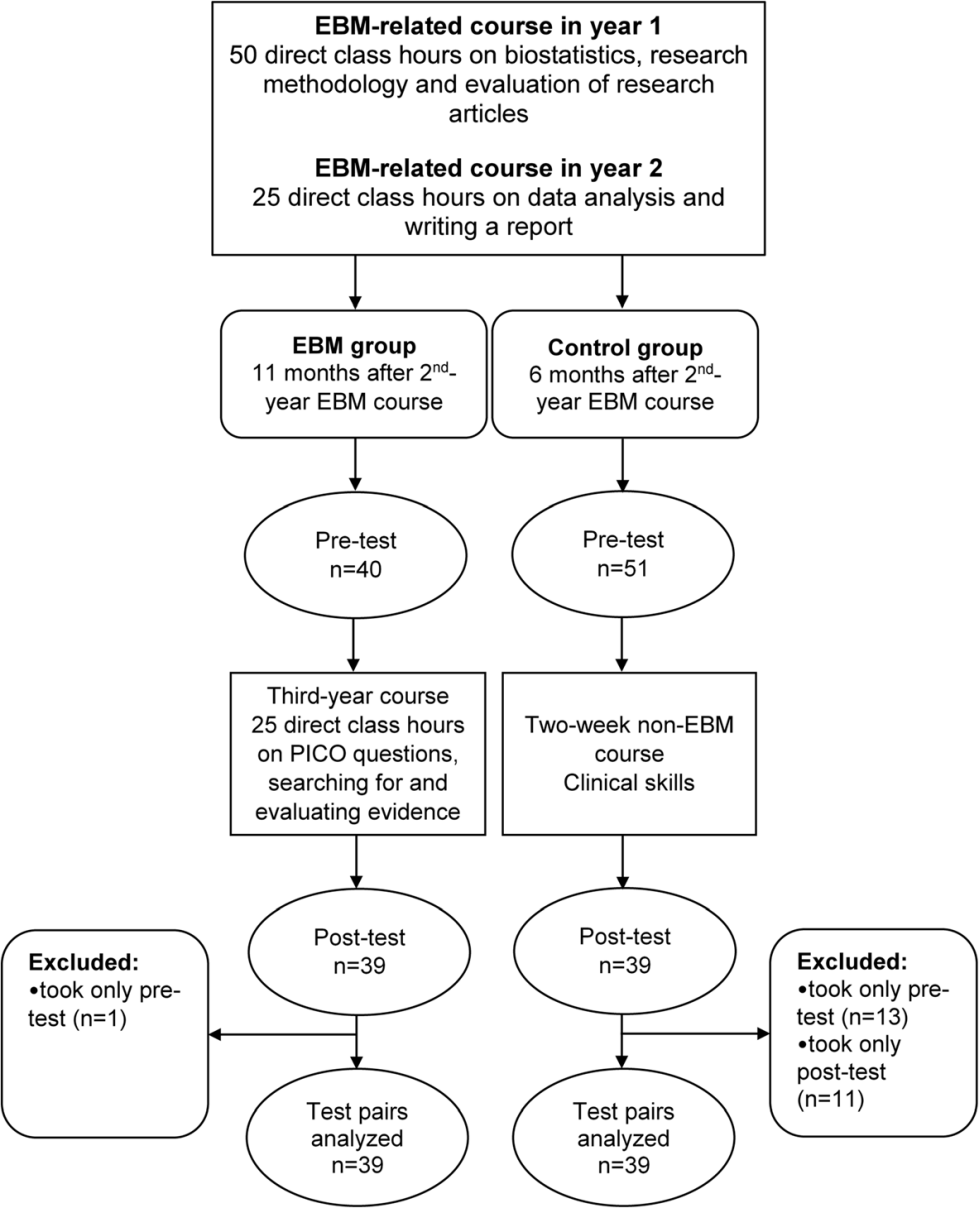
Flow of the participants in the study

At the USSM, EBM is a part of a vertically integrated course on research methodology, consisting of three separate courses held during the first 3 years of a 6-year medical graduate program taught by experienced researchers in the field of biomedicine [20, 21]. In brief, first-year students attend 2 weeks (50 direct class hours) of face-to-face lectures, seminars and practical exercises on biostatistics and research methodology. The competencies gained after this first-year course are the basic understanding of research methodology in medicine, critical evaluation of scientific reports, and understanding and application of basic biostatistics. In the second year, students attend 1 week of face-to-face practical exercises (25 direct class hours) in which they apply the knowledge gained in the first year to analyse a dataset from published research studies and write a brief research report. In the third year (25 direct class hours), the students are trained in EBM steps, including formulating the PICO (problem, intervention, comparison, outcome) question, searching for evidence and critical evaluation of evidence related to specific clinical problems (Fig. 1).

### Participants

Study participants were third-year medical students (in further text the EBM group) at the USSM. They took all three EBM tests during pre- and post-course assessments. We also tested second-year medical students, who served as a control for test-retest effects of the EBM group, due to the fact that the same tests were used for pre- and post-course assessment (Fig. 1). Both groups passed the two previous research methodology courses in their first and second study years. The EBM group included third-year students who were tested pre- and post-course in November 2015, 10 months after they took the second research methodology course. The Control group included second-year students, who were tested pre- and post-course in July 2016, 7 months after they passed their second research methodology course. The testing of the Control group was performed during a non-related course (the Clinical Skills II course). Due to the timing of the courses during which the testing was implemented (the end of the second and the start of the third academic year), both student groups not only had the same baseline amount of research methodology teaching but they also attended the same number of courses except a single course (Microbiology and Parasitology course was attended by the EBM group only, and does not contain any elements of EBM teaching).

### Procedure

The EBM group was pre-tested on the day students started their 1-week EBM course, and tested again 1 day after the course. Similarly, the Control group was tested on the first day of their non-EBM 2-week course, and then again immediately after the end of the course. The tests were paper-based and the students were allowed to use calculators. We also collected demographic and academic achievement data: gender, age, overall grade point average from the previous academic year (GPA, on a range from 2 – pass to 5 – outstanding), and grades from two previous research methodology courses. In order to ensure participants’ anonymity but allow the pairing of the pre- and post-test results, each participant wrote a 6-element code on the first page. The time allowed for the completion of all three tests was 2 h. Both groups were introduced to all three tests, but due to the change in the teaching schedule, the Control group was only handed the ACE and Berlin tests but not the Fresno test, which requires significantly more time for completion than the other two tests. As the Control group was included in the study to control the effectiveness of educational intervention (by assessing the test-retest effects, and by controlling for the effect of guessing on binomial/multiple choice tests), the introduced change was acceptable.

Because the EBM group was tested with three tests, one of which took substantially longer time than the others, there was a possibility of respondents’ fatigue. Our assumption was that if the test sequence was universal for all participants, there would be a significant dropout in answer frequency on whichever test was the last one. To eliminate that bias, the test sequence for the EBM group was randomized using a web-based random number generator web-application (www.randomization.com) to create three different combinations of the tests’ sequence. The Control group received the ACE and Berlin tests, in that order. Randomization here was not implemented because fatigue was not expected as the tests lasted 20 min at most. The participation of students was voluntary and all participants received a small token for their participation (USB memory stick or a chocolate bar). The study was approved by the Ethics Committee of the USSM.

### Instruments

We used three validated and commonly used EBM tests that had three different answering formats: the ACE (Assessing Competency in EBM) Tool with binary answering format [14] (ACE test), the Berlin Questionnaire with multiple-choice format [11] (Berlin test) and the Fresno test with open-ended answers [10]. All instruments were translated into Croatian by the researchers and then back-translated into English by two independent translators, a professional translator and one of the researchers (IB). Both back-translations were cross-checked by two senior researchers (AM and MM) and revised accordingly. This procedure resulted in the correction of two statements for clarity.

The ACE test is a 15-item instrument that assesses EBM knowledge in four different domains: question formulation, literature search, appraising the evidence and applying the evidence [14]. A test-taker reads a patient scenario and answers 15 Yes/No questions. The total score is the sum of all correct answers, ranging theoretically from 0 to 15 points.

The Berlin test is a 15-item, multiple choice instrument on a scale from 0 to 15 points. The questions (on formulating an answerable clinical question, appraisal of evidence, consideration of clinical decision options) are built around hypothetical clinical scenarios and linked to published articles. A test-taker has to read a clinical scenario and then choose the correct answer among five offered answers. We used the A version of the test [11].

The Fresno test is an 18-item instrument, consisting of open-ended questions which are rated according to one of four grade categories: “not evident”, “minimal”, “strong” and “excellent”. The total score ranges from 0 to 124 [10]. The questions are formulated as scenarios where respondents must formulate a clinical question and continue with the appraisal of evidence, identify the most appropriate research design to answer a research question, demonstrate knowledge of electronic database searching, identify the issues regarding the validity and relevance and discuss the magnitude and importance of relevant research. Compared to the ACE and Berlin tests, the Fresno test constitutes a different type of assessment where the participant is placed in a problem-based situation instead of choosing one of the offered answers, as it is the case with the other two tests. For the purpose of this study, we used the scenarios from the original instrument, translated into Croatian (3).

EBM topics covered by the three EBM knowledge tests [14, 22] are shown in Table 1.

**Table 1:** Evidence-based medicine (EBM) topics addressed by questions in three EBM tests^a^

Topics	No. of test questions covering a topic		
	ACE test	Berlin test	Fresno test
Asking questions^b^	1, 2		1a, 1b
Information sources^b^			2
Study design^b^		6, 7, 14	4, 11, 12
Searching for evidence^b^	3, 4		3
Internal validity^b^	5, 6, 7, 8, 9, 10, 11	8, 10, 15	6
Magnitude of effect/ clinical importance		4, 5, 9, 11	7, 9a, 9b, 9c, 10
Application	12, 13, 14, 15	13	5
Diagnostic accuracy		1, 2, 3, 12	8a, 8b, 8c, 8d, 8e

^a^Each question is numbered according to its position in the original test. The ACE (Assessing Competency in Evidence-based Medicine) test has 15 dichotomous (Y/N) questions; each correct answer is awarded with one point [14]. The Berlin test has 15 multiple choice questions with five options and a single correct answer; each correct answer is awarded one point) [11]. The Fresno test has 19 open-ended questions, where each question is awarded a different number of points. In order to calculate discrimination and difficulty index for this test, answer for each participant is divided by the maximum number of points for that question [10]. The scenarios from the original instrument were translated into Croatian for this study

^b^EBM topics covered in the third-year EBM course at USSM

Test scores for all three tests were assessed by one author (IB) and checked by another (AM); there were no inconsistencies between their assessments.

### Statistical analysis

Absolute numbers and percentages were used to describe gender distribution in the EBM and Control group. Other baseline characteristics and test scores were presented as group medians and interquartile ranges (IQR). Distributions of these variables were tested for normality with Shapiro-Wilk test. Distribution of random scores on the ACE and Berlin tests were calculated using the binomial distribution.

The effect size of educational intervention was expressed as a median and associated 95% confidence interval (95% CI) of individual changes in test scores from pre- to post-test, with changes expressed either in absolute scores, or in percentages of a maximum possible score. The differences between pre- and post-test scores in each study group were tested using the Wilcoxon signed-rank test, whereas the differences in test scores between the groups were assessed using the Mann-Whitney U test. Individual scores on three EBM knowledge tests were expressed as percentages of the maximum possible score and compared between the tests using the Friedman test followed by post-hoc Wilcoxon signed-rank test. The scores on a particular EBM topic were expressed as percentages of the maximum possible score.

We also compared average item difficulty indices of the tests. The post-test data for the EBM group were used to calculate the difficulty index (DI) for each item on the ACE and Berlin tests, and average percent of maximum score per item on the Fresno test. The same data were used to assess internal consistency of scores for each test by calculating Cronbach’s alphas.

We used a significance level of α = 0.05. Statistical analysis was performed using SPSS Statistics for Windows, Version 19.0 (IBM Corp., released 2010, Armonk, NY, USA).

## Results

### Baseline characteristics

Out of 135 eligible students (45 from the EBM group and 90 from the Control group), 91 students took part in the study (67% response rate). Sixty-seven students completed both the pre- and post-course tests (Fig. 1). The reasons for dropouts were academic obligations of the second-year students on the testing day. The baseline characteristics of students from both groups are presented in Table 2.

**Table 2:** Basic characteristics of the study participants

Characteristics (median and IQR, or No. and %)	Control group (*n* = 28)	EBM group (*n* = 39)
Age (years)^a^	20 (20–21)	21 (20–21)
Female gender (No.)	23 (82%)	25 (64%)
Grade point average^b^	4.0 (3.5–4.0)	3.7 (3.3–4.1)
Grade of EBM I course^b^	5.0 (4.0–5.0)	5.0 (4.0–5.0)
Grade of EBM II course^ba^	5.0 (4.0–5.0)	5.0 (5.0–5.0)

^a^Significant differences between the groups at the 0.05 level

^b^In the Croatian education system, passing grades range from 2 (poor) to 5 (outstanding), 1 is failure. EBM group – the experimental group of students taking the Evidence Based Medicine (EBM) course in year 3 of the curriculum, IQR – interquartile range, EBM I and II course – EBM-related courses in year 1 or 2 of the medical curriculum, respectively. Distribution of gender in the groups was similar to that of the USSM student population [34]

### Effect of EBM intervention

After a week-long EBM course, students scored significantly higher than baseline on all three EBM knowledge tests (Table 3, *P* < 0.001 for all). The largest improvement in EBM knowledge from the pre-test was achieved on the Fresno test, whereas the improvements in ACE and Berlin test scores were comparable (Table 3). Namely, post-test percent increase in the Fresno test scores was significantly higher than corresponding values on either the ACE or Berlin test (Friedman test for related samples, *P* = 0.001; post hoc tests *P* ≤ 0.010), while no difference was found between the ACE and Berlin tests (post hoc test, *P* = 0.999). Regarding the estimates of post-test EBM knowledge expressed as percentage of maximum possible score on a scale, the tests also substantially differed (Friedman test, *P* < 0.001; post hoc tests *P* ≤ 0.036). On average, students scored highest on the ACE test (median percentage of maximum possible score 73, 95% CI 67–80%), than on the Berlin test (60, 95% CI 47–67%), and lowest on the Fresno test (45, 95% CI 40–50%) [23].

**Table 3:** Test scores on three evidence-based medicine (EBM) knowledge tests measured before (pre-test) and after (post-test) the educational intervention in the EBM group

EBM knowledge test (min-max score)	Pre-test	Post-test	*P*-value^*^	Improvement from the pre-test
	Median score (95% confidence interval)			Percentage of the maximum possible score (range)
Berlin test (0–15)	6 (5–7)	8 (8–10)	< 0.001	13% (7–20%)
ACE test (0–15)	8 (8–9)	11 (11–12)	< 0.001	13% (13–20%)
Fresno test (0–124)^a^	11 (10–19)	56 (51–63)	< 0.001	31% (25–42%)

^*^Wilcoxon signed test

^a^*n* = 35 for the Fresno test (see Fig. 1)

The effectiveness of EBM education was further confirmed by comparison with the Control group. While groups were comparable at baseline in assessed level of EBM knowledge on the Berlin and the ACE tests, after the intervention, only the EBM group showed significant increase in knowledge on both tests (Additional file 1: Table S1). In the Control group, post-test scores on either the ACE or Berlin tests did not significantly change from the pre-test scores, confirming that the increase observed in the EBM group was indeed the effect of intervention (Additional file 1: Table S2).

### Test score distributions and average item difficulty

While we found significant differences in median percentages of the maximum possible scores between all three EBM tests in the intervention (EBM) group, the shape of post-test score distributions and average item difficulty of these tests also indicated that they may also be differentiated according to their potential to discriminate between the levels of EBM knowledge. In particular, individual percentages of maximum possible-scores on the ACE test were differently distributed than the corresponding scores on the Berlin or Fresno tests (Additional file 1: Figure S1, Friedman test of *P* < 0.001 with post hoc analysis of *P* ≤ 0.002). Specifically, ACE test scores grouped at the high end of the score scale that ranged from 0 to 100%, whereas the groupings of Berlin and Fresno test scores were much more dispersed. The percentages of maximum possible-scores on the Fresno test were on average lower than the scores on the Berlin test (Additional file 1: Figure S1, Friedman test of *P* < 0.001 with post hoc test of *P* = 0.036).

Item difficulty analysis confirmed the clustering of post-test scores on the ACE test, as 7 (47%) of ACE test items exhibited the difficulty index (DI) of ≥0.9 (i.e., more than 90% of students correctly responded to an item) compared to just 1 (6%) of the items on the Berlin test. Difficulty indices for the ACE and Berlin tests and average percent-of-maximum-possible-score for items on the Fresno test are shown in Additional file 1: Table S3.

### EMB topics covered by EBM knowledge tests

When we analyzed which EBM topics (see Table 1) were associated with the most difficult items in the EBM group at post-test, the tests differed in what appeared to be the most difficult topic (Additional file 1: Table S3). Whereas ACE test results identified *Internal validity* and *Application* as such topics, items associated with these topics on the Berlin test all had the satisfying difficulty indices that could discriminate participants with low and high total scores (DI ≥ 0.49). In the Fresno test, an *Application* item also exhibited satisfying difficulty (47% average percent of maximum possible scores). As for the *Internal validity* topic in the Fresno test, although the assigned item was identified as hard to solve (26% average percent of maximum possible scores) it was far from being the “most difficult”. The “most difficult” topic in the Berlin test was *Diagnostic Accuracy*, while *Diagnostic accuracy*, *Clinical Importance*, *Study Design* were the most difficult topics for the Fresno test.

Similarly, when we compared EBM topic-specific percentage of maximum possible scores between different knowledge tests, we found significant differences between the Fresno and other two tests. Whereas the distributions of these percentage scores where comparable between the three tests on EBM topics *Study design*, *Clinical importance*, and *Applicability* (Table 4, Wilcoxon signed rank test or Friedman test, *P* ≥ 0.163), Fresno test scores were significantly lower than those of the other two tests on the topics of *Internal validity*, *Diagnostic accuracy*, and *Searching* (Table 4, Wilcoxon signed rank tests or Friedman test of *P* < 0.001 for all with post hoc analysis, *P* ≤ 0.001 for all). The only topic for which the Fresno test showed higher scores was the topic of *Asking questions* (Table 4, Wilcoxon signed rank test for comparison with the ACE test, *P* = 0.002). In contrast, the ACE and Berlin tests, which could be compared only on the topics of *Internal validity*, and *Application*, exhibited comparable percent score distributions (Table 4, Friedman test *P* ≥ 0.668).

**Table 4:** Percentage of maximum-possible-score (median, 95% confidence interval) for specific evidence-based medicine (EBM) topics in the EBM group after the intervention and associated percent change from the baseline (*n* = 39^a^)

EBM topic	ACE test			Berlin test			Fresno test		
	Max score	Post-test (% of max score)	Improvement from pre-test (% of max score)	Max score	Post-test (% of max score)	Improvement from pre-test (% of max score)	Max score	Post-test (% of max score)	Improvement from pre-test (% of max score)
Asking questions	2	100 (100–100)	50 (0–50)				6	100 for all students – no variability	33 (33–50)
Information sources							6	33 (33–67)	33 (0–33)
Study design				3	67 (67–67)	0 (0–33)	20	55 (35–80)	30 (20–60)
Searching for evidence	2	100 (100–100)	0 (0–50)				8	75 (38–75)	38 (38–63)
Internal validity	7	71 (71–71)	14 (14–29)	3	71 (61–81)	0 (0–33)	24	21 (21–21)	21 (0–21)
Clinical importance				4	50 (50–50)	0 (0–25)	28	46 (43–60)	43 (32–46)
Application	4	50 (50–75)	0 (0–25)	1	100 (0–100)	0 (0–0)	12	42 (42–75)	42 (42–42)
Diagnostic accuracy				4	50 (25–50)	25 (25–25)	20	0 (0–20)	0 (0–20)
All topics	15	73 (67–80)	13 (13–20)	15	60 (47–67)	13 (7–20)	124	45 (40–50)	31 (25–42)

^a^*n* = 35 for the Fresno test (see Fig. 1)

With regard to an EBM topic-specific changes in percentage of maximum scores from the baseline, we observed significant increases in knowledge after the EBM course on all tests and on almost all EBM topics (Table 4, one-sample Wilcoxon signed rank test for change, *P* ≤ 0.027). In the ACE and Berlin tests, the only EBM topic on which educational intervention had apparently no effect was *Application* (Table 4, one-sample Wilcoxon signed rank test for change, *P* ≥ 0.169). The opposite finding was true for the Fresno test, where the largest increase in percentage scores compared to baseline was observed for the *Application* topic. As the post-test knowledge assessment on the *Application* topic was comparable between three tests, this discrepancy must stem from the pre-test students’ answers. The questions on the ACE and Berlin tests were of the closed type (binary or multiple choice, respectively) and could “guide” an unprepared student to choose the correct answer simply by offering limited options to select from, serving as a reminder of the best answer or being the result of chance (on average50% for binary and 20% for multiple answer choices). In contrast, the Fresno test has open-ended questions that do not allow for chance guessing or guidance to the correct answer.

### Heterogeneity of items on EBM knowledge tests

Whereas the reliability of the Fresno test for the post-test results of the EBM group was good (Cronbach’s α = 0.75), that of the ACE test, measured in the same group and at the same time point, was very low (Cronbach’s α = 0.13). Although some level of underestimation of Cronbach’s α could be expected due to dichotomous items on the ACE test (as correlations among dichotomous items tend to underestimate true correlations), such low value of α additionally points towards the presence of heterogeneous items on a scale. Because of sample size and item-number limitations, we could not perform the factor analysis or the analysis of consistency of items per specific EBM topic to identify multiple factors/traits underlying the ACE test items. Additionally, if a group was predominated by low achievers, the low reliability on a knowledge test could also be affected by a random component – a random choice of answers (in such case the test scores would not reflect the knowledge of EBM but the random distribution of guessed answers) [24]. This, however, was not the case in our study as we showed that both the EBM and Control groups exhibited the level of EBM knowledge that was satisfactory for their EBM education level [14], with the average ACE test score for both pre- and post- testing conditions being significantly higher than expected for random choosing of answers (Table 2, *P* < 0.001 for all Mann-Whitney tests comparing random scores to students’ scores; see also Additional file 1: Figure S2).

The reliability of the Berlin test (Cronbach’s α = 0.63) was higher than that of the ACE test. Although this value is acceptable [25], it is close to the acceptance threshold, suggesting the presence of heterogeneous items on the Berlin test, too. Similar to the ACE test, we showed that the level of EBM knowledge measured on the Berlin test improved after the course, as Berlin test scores were also significantly higher than those expected if the students randomly chose the answers (Table 3, *P* < 0.001 for all Mann-Whitney tests comparing the random Berlin test scores to students’ scores; see also Additional file 1: Figure S3).

## Discussion

Our study showed significant differences between the ACE, Berlin and Fresno tests in a group of third year medical students after a 25-h EBM course. Although the knowledge scores improved in the intervention (EBM) group on all three tests after the course, both when compared with their pre-test results and the Control group, the post-test scores and the magnitude of improvements substantially differed between the tests. Students achieved the highest scores on the ACE test, followed by the Berlin test and scored lowest of the Fresno test. This meant that, after having approximately 75 h of direct class teaching in research methodology and statistics during the first 2 years of medical study, and after 25 class hours of the EBM course all students passed the ACE test according to the 50% cut-off of the maximum attainable score [14]. Median ACE test score indicated an average knowledge of EBM concepts. The same level of knowledge was observed by ACE test creators in medical trainees with advanced EBM knowledge [14], whereas the scores observed in medical students taking their first clinical year of EBM training [15] were somewhat lower than in our study. This is consistent with the fact that our students were introduced to some aspects of EBM during the first and second year of their studies and actually completed their undergraduate EBM training after the third year.

At the same time, after gaining the same amount of knowledge during the intervention, our students scored worse on the Berlin test, in which questions are built around clinical scenarios. In comparison to other students with apparently similar level of medical knowledge, the level of EBM knowledge assessed on the Berlin test in our sample was somewhat higher than was reported for the final year Malaysian students (mean of 45%) [16], and was comparable to Australian third-year medical students, who were enrolled in the first year of clinic-based training and first year of formal EBM training (55%) [15], and to Syrian medical students with mixed educational levels from the first to the sixth year (55%) [12].

Finally, students’ median score on the Fresno test was low: 45% (95% CI 40–50%), which suggests unsatisfactory understanding of the subject matter involved. Compared to the results of other medical students, such low Fresno test score was similar to average score observed for the final year Malaysian medical students (mean of 45%) [16], or for undergraduate physiotherapy students who had their EBM teaching verticalised throughout the first 3 years (55%) [26]. Somewhat higher median score on the Fresno test (60, 95% CI 60–64%) was observed for fifth-year medical students from Jordan, whose clinical experience from family medicine rotations was at the level that might have influenced their knowledge and perception of EBM concepts [27].

We also observed significant differences between the tests in pre-post knowledge improvements, where Fresno test results were significantly higher compared to scores on the ACE and Berlin tests, indicating that the assessment of the accumulated level of students’ knowledge during the course would be influenced by the choice of the EBM test. Further, the ACE test exhibited lower potential to discriminate between the levels of EBM knowledge as compared to other two tests. Last but not the least, the average difficulty of items assigned to a particular EBM topic was inconsistent between the tests. Specifically, we observed significant differences in the distribution of EBM topic-specific percentages of maximum possible scores between the Fresno, ACE and Berlin tests, and have identified that items assigned to the same EBM topic but on different EBM knowledge tests exhibited variable levels of difficulty.

While the latter findings suggest between-the-tests heterogeneity of questions that are designed to measure the level of knowledge on an entire EBM domain, there are additional factors which should be taken into account when discrepancies between the tests in the assessed level of knowledge for the same participants are considered. The first factor is the difference in the educational targets of these tests, more specifically the difference between a performance-based assessment tool and a simple knowledge test. While the ACE test directly assesses the knowledge, the Berlin and Fresno tests, which are built around clinical scenarios, attempt to assess a subject’s ability to apply their knowledge in the context of actual practice/problem solving. The second factor is the type of test questions. The ACE test uses a dichotomous question type, where the participant who does not know the answer still has, on average, a 50% chance of guessing the true answer. In such circumstances, a student who randomly chooses all the answers could on average score 7.5 points on the 15-point ACE test scale, which may affect the reliability of the test (for example, when students with poor academic performance prevail in a class). In the Berlin test, a participant has to choose one out of five different options, having a 20% chance for guessing the right answer by chance, making the effect of random guessing on the Berlin test much smaller. Finally, the Fresno test consists of open-ended questions where there is no possibility of choosing the right answer by random, and therefore, when presented with educational intervention, the participant may show significantly higher improvement compared with other two tests because its baseline is not confounded with random guessing. Therefore, the underlying reason for the greatest improvement on the Fresno test may be results of different test type. The third factor is related to the content of different measures. Although all three tests were designed for EBM knowledge assessment, and therefore all should measure similar constructs, the Fresno test, which has clinically integrated scenarios, encompasses a wider area of EBM knowledge than the Berlin test, which is focused on concept recognition [28]. Furthermore, while the Fresno test covers the whole range of EBM areas, the ACE and Berlin tests are more specifically focused on certain areas. For family medicine residents, the Fresno test emerged as the best option in a systematic review assessing the validity of EBM measures [28]. The downside of the Fresno test is that it examines each of the four different areas with a single question, which may be insufficient for a comprehensive knowledge assessment. A recent systematic review emphasized that the Berlin test assesses specific skills, such as developing a clinical question or a search strategy, while the Fresno test requires the demonstration of knowledge and skills across four steps of evidence-based practice applied to clinical scenarios in an open-ended format, which potentially requires higher learning levels which differentiate from basic content understanding and concept recognition [29]. Evidently, although all three measures are standardized measures of basic EBM knowledge, they do not produce similar results in testing EBM skills in the same population. This means that there should be either a standardized EBM test to measure students’ learning progress across the medical curricula or a careful choice of an EBM test that best suits the curriculum in question.

The largest relative result (comparing the average result with the maximum possible test score) and the clustering of scores on a high end of the scale was achieved on the ACE test, followed by the Berlin and Fresno tests, whose scores were more widely dispersed. Whether this difference stems from the difference between a criterion-referenced test [30], whose goal is to make a YES/NO decision about whether an examinee demonstrates proficiency in the content and competencies in an area, and a norm-referenced test, which is intended to rank the entire set of examinees in order to make comparisons of their performances relative to one another, remains to be seen. Nevertheless, given the effect of random guessing on ACE test scoring, very low Cronbach’s alpha recorded for this test in our study, and the fact that the test was not stringently validated in the original publication [14], we cannot eliminate the possibility that the highest scores on the ACE test in our study was just an artefact of the test’s unreliability/invalidity. Namely, while the authors of the ACE test claimed to have validated the test, they actually reported only the finding of significant differences between novice, intermediate, and advanced EBM students, which in reality amounted to less than a single correct answer on the ACE score scale. Overall, based on what we observed in this study, we can state there is a clear difference in the educational targets for the three tests, which may affect the suitability of the test in a particular target population: the Fresno and Berlin tests are more suitable for more experienced students with clinical experience and the ACE test might be more suitable for students in preclinical years [28]. In order to assess the level of knowledge in a specific EBM domain, the choice of the test should be based on the course design, content and learning objectives.

The largest effect size of EBM educational intervention was found on the Fresno test, where the results dramatically improved in the EBM group when compared to the pre-test score. However, post-test results were lower than the results from other studies on this test in student populations [13, 19] and may be related to the fact that the EBM course in our study was not embedded or related to clinical courses or clinical experience, which predominate in the Fresno test. This also means that a standalone EBM course related to research methodology and biostatistics can improve the knowledge of EBM to a certain point, but clinical integration could be instrumental for most effective delivery of EBM education.

Our study had several limitations. The sample size was small in both groups, calling for replication in larger sample sizes. Furthermore, we simultaneously applied three knowledge tests in the EBM group, so that the results could have been affected by the fatigue of the participants. To eliminate that factor, the Control group was tested only with the Berlin and ACE tests and the tests were randomized in the individual test packages for the EBM group. However, there was no control group for the Fresno test, and although the EBM group was better in the ACE test and the Berlin test when compared to the Control group, we cannot claim that the same group would have been better on the Fresno test, due to significant differences that were identified between the three tests. Also, we assessed students’ EBM knowledge 1 day after the intervention. Future studies should focus on longer-term knowledge assessment (e.g. several months after the intervention) in order to assess the retention of knowledge. Finally, we used only three most frequently used measures for EBM knowledge assessment, and other possible measures [31] were not included in the study.

## Conclusions

Despite its limitations, our study demonstrated that the choice of EBM knowledge test for evaluating EBM training of medical students affects the estimation of EBM knowledge and intervention effect. As different tests focus on different EBM content, test scores then reflect the content and learning exposure more than students’ innate ability and experience. This has important implications for EBM training: assessment measures should be customized for a specific EBM training target population, and preferably use measures that assess both the previously acquired knowledge and further application of that knowledge. The latter is particularly important in view of the reports that the application of knowledge in a new context is impaired if individuals have not learned the basics of the topic [32]. Based on what was presented in this study, we suggest that, in the majority of educational settings, the Berlin test is preferred over the ACE test. As for the Fresno test, which is the most discriminative test but needs the most effort and investment by the teacher, it is probably best suited for smaller groups or smaller classes. However, there is no single tool developed for assessing the higher dimensions of EBM knowledge according to Bloom’s taxonomy of educational objectives [32, 33].

## Additional file

Additional file 1: This additional file contains more details on the distributions of the test scores and differences in the control and intervention groups. (DOCX 102 kb)

## Abbreviations

ACE: Assessing competency in evidence based medicine tool
CI: Confidence interval
EBM: Evidence based medicine
GPA: Grade point average
IQR: Interquartile range
SPSS: Statistical package for social sciences
USSM: University of Split School of Medicine
