# Supplementary material for: How to choose an evidence-based medicine knowledge test for medical students? Comparison of three knowledge measures
Source: BMC Med Educ. 2018 Dec 4;18:290. doi: 10.1186/s12909-018-1391-z (PMC6278026; doi:10.1186/s12909-018-1391-z)

**Additional file 1**

**Table s1.** Comparison of test scores on the Berlin and ACE knowledge tests between the EBM and the control group of students

| **EBM knowledge test (min-max score)** | **Pre or post-test** | **Control group** | **EBM group** | **Median difference (95% CI), P-value*** |
| --- | --- | --- | --- | --- |
|  |  | Median test score (95% CI) | |  |
| Berlin test (0-15) | Pre-test | 6.0 (5.0–7.0) | 6.0 (5.0–7.0) | 0.0 (-1.0, 1.0), P=0.607 |
|  | Post-test | 6.0 (6.0–8.0) | 8.0 (8.0–10.0) | 2.0 (1.0, 3.0), P=0.001 |
| ACE test (0-15) | Pre-test | 9.0 (9.0–10.0) | 8.0 (8.0–9.0) | -1.0 (-1.0, 0.0), P=0.239 |
|  | Post-test | 9.0 (9.0–10.0) | 11.0 (11.0–12.0) | 2.0 (1.0, 2.0), P<0.001 |

EBM – evidence-based medicine, CI – confidence interval

*Mann-Whitney test

**Table s2.** Comparison of test scores on the Berlin and ACE knowledge tests in the control group of students

| **EBM knowledge test (min-max score)** | **Pre-test** | **Post-test** | **Median difference (95% CI), P-value*** |
| --- | --- | --- | --- |
|  | **Median test score (95% CI)** | |  |
| Berlin test (0-15) | 6.0 (5.0–7.0) | 6.0 (6.0–8.0) | 0.0 (-1.0, 1.0), P=0.693 |
| ACE test (0-15) | 9.0 (9.0–10.0) | 9.0 (9.0–10.0) | 0.0 (-1.0, 1.0), P=0.919 |

EBM – evidence-based medicine, CI – confidence interval

*Wilcoxon Signed Rank test

**Table s3.** Difficulty indices and average percent-of-maximum-possible-score for items in three EBM tests.

| **ACE** | | **Berlin** | | **Fresno** | |
| --- | --- | --- | --- | --- | --- |
| **Item No** | **Difficulty index** | **Item No** | **Difficulty index** | **Item No** | **Average percent-of-maximum-possible-scores** |
| **1** | 0.87 | **1** | 0.31 | **1a** | 1.00 |
| **2** | 0.87 | **2** | 0.67 | **1b** | 1.00 |
| **3** | 0.92 | **3** | 0.13 | **2** | 0.56 |
| **4** | 0.85 | **4** | 0.95 | **3** | 0.59 |
| **5** | 1.00 | **5** | 0.38 | **4** | 0.63 |
| **6** | 0.03 | **6** | 0.64 | **5** | 0.47 |
| **7** | 0.92 | **7** | 0.72 | **6** | 0.26 |
| **8** | 0.97 | **8** | 0.72 | **7** | 0.38 |
| **9** | 0.90 | **9** | 0.28 | **8a** | 0.34 |
| **10** | 0.97 | **10** | 0.49 | **8b** | 0.14 |
| **11** | 0.28 | **11** | 0.31 | **8c** | 0.14 |
| **12** | 0.79 | **12** | 0.46 | **8d** | 0.03 |
| **13** | 0.97 | **13** | 0.56 | **8e** | 0.11 |
| **14** | 0.21 | **14** | 0.51 | **9a** | 0.94 |
| **15** | 0.28 | **15** | 0.87 | **9b** | 0.57 |
|  |  |  |  | **9c** | 0.86 |
|  |  |  |  | **10** | 0.31 |
|  |  |  |  | **11** | 0.29 |
|  |  |  |  | **12** | 0.80 |

**Figure s1.** Distributions of percentages of maximum scores obtained on three EBM knowledge tests after an educational intervention in the EBM group of students.


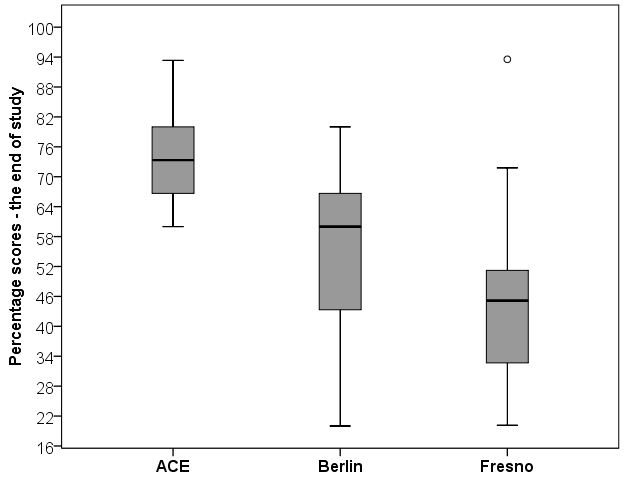


**Figure s2.** ACE test score distribution expected under random choice, and observed in tested students before and after the educational intervention, shown by the study year. Brackets mark statistically significant differences in ACE scores between the random choice result and scores observed in student groups as determined by Mann-Whitney testing (full line – significance at 0.5 level, dashed line – 0.1 level).


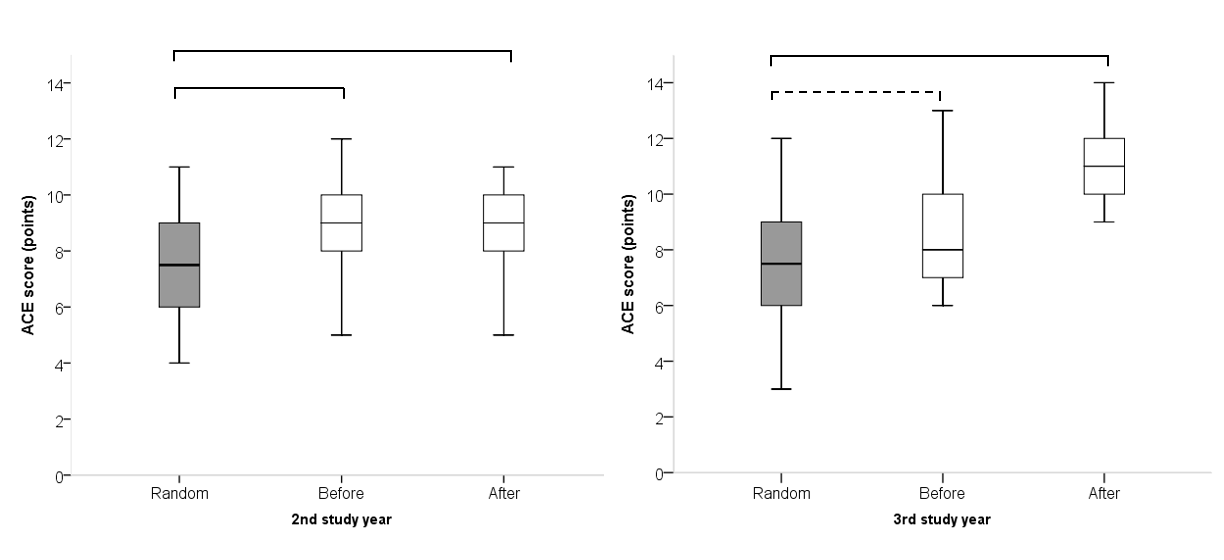


**Figure s3.** Berlin test score distribution expected under random choice, and observed in tested students before and after the educational intervention, shown by the study year. Brackets mark statistically significant differences in Berlin scores between the random choice result and scores observed in student groups (full line – significance at 0.5 level).


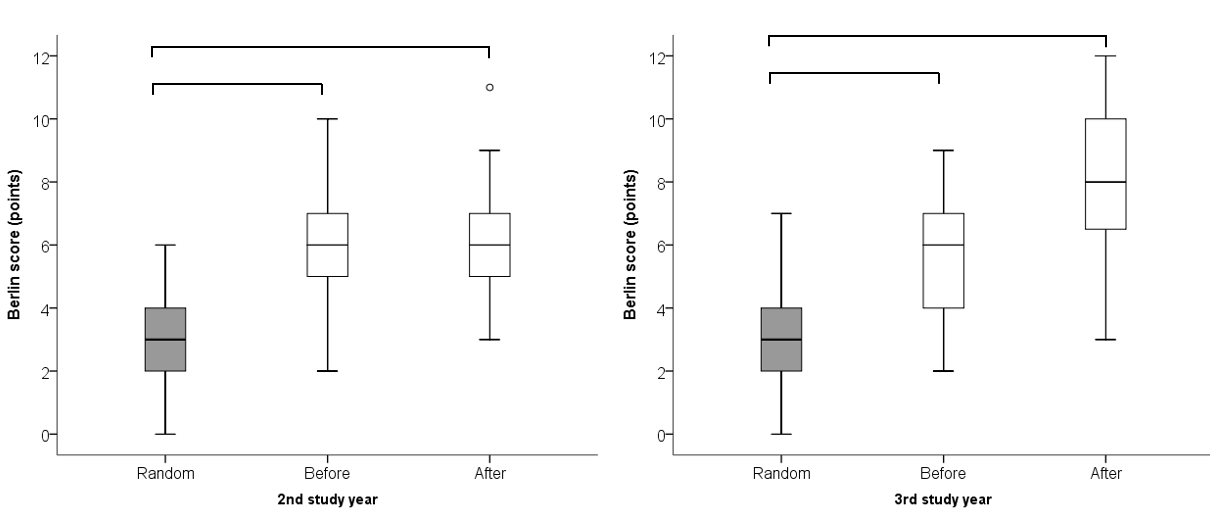

Supplement: Supplementary file 1 — This additional file contains more details on the distributions of the test scores and differences in the control and intervention groups. (DOCX 102 kb) [file 12909_2018_1391_MOESM1_ESM.docx]
